# Special Issue IJMS—Molecular Mechanisms of Diabetic Kidney Disease

**DOI:** 10.3390/ijms25020790

**Published:** 2024-01-08

**Authors:** Ligia Petrica

**Affiliations:** Centre for Molecular Research in Nephrology and Vascular Disease, Department of Internal Medicine II, Division of Nephrology, “Victor Babeș” University of Medicine and Pharmacy, 300041 Timișoara, Romania; petrica.ligia@umft.ro

Diabetic kidney disease (DKD), as a major microvascular complication of both type 1 and type 2 diabetes mellitus (DM), accounts for over 40% of patients that reach end-stage renal disease and are referred to renal replacement therapies [1]. The tubulocentric concept with regard to DKD has emphasized the pivotal role of the proximal tubule and of the tubulointerstitial compartment in the development of DKD. The glomerular theory has raised similar interest, with a special focus on the contribution of podocyte injury in the course of DKD. Chronic systemic inflammation and the role of the inflammatory response in the development and progression of chronic kidney disease and DKD have been recognized as being highly important. The inflammatory response, which involves the innate immune system, mitochondrial dysfunction, as well as epigenetic mechanisms, plays an important role in the development of albuminuria in the course of type 2 DM. Specific molecular signatures and epigenetic profiles have been discovered that underscore the complexity of DKD [2,3].

The hypoxia-inducible factors (HIFs) HIF-1, HIF-2, and HIF-3 are the main mediators of the metabolic responses to the state of hypoxia, which is assumed to be a mechanism of major import in the occurrence and progression of diabetic kidney disease (DKD) [4,5].

*Stanigut AM*, et al. provide a compelling review addressing the direct and indirect role of deregulated HIF-1 activity in the pathogenesis of DKD. The authors provide data concerning the recent findings of research in the field of hypoxia and the implications of HIF-1 in the initiation and development of DKD at both the glomerular and tubular level. The interaction of HIF-1 with other nonhypoxic factors, such as angiotensin II, is also discussed. Moreover, the authors reveal the dual activity of HIFs as pro-fibrotic or anti-fibrotic factors and the roles of HIF-1 and HIF-2 in the glomerular endothelium abnormalities seen in DKD. Of interest, a new mechanism of HIF-1 protection against hyperglycemia-induced oxidative injury and the enhancement of mitophagic activity, at least partially via the Parkin/PINK1 signaling pathway in tubular cells, is discussed. In their review, the authors emphasize the clinical impact of therapies involved in modulating HIF-1 activity, such as sodium-glucose cotransporter 2 inhibitors (SGLT2i) and oral hypoxia-inducible factor (HIF) prolyl hydroxylase inhibitors [6].

The imbalance between the oxidative stress and antioxidant defense mechanisms represents one of the major phenomena involved in the structural and functional changes that occur during the course of DKD. In their study, *Andrade-Sierra J and co-workers* addressed the multiple mechanisms by which oxidative damage and inflammation contribute to the pathogenesis of DKD in type 2 DM patients. An analytical cross-sectional study was carried out in patients with early-stage CKD (1, 2, 3) with and without T2DM. The levels of the pro-inflammatory cytokines IL-6 and TNF-alpha, lipoperoxides (LPO), nitric oxide (NO), and the superoxide dismutase activity (SOD) were assessed in all the patients. Also, glutathione peroxidase (GPx) and the total antioxidant capacity (TAC) were determined. They found a decrease in Hb and an increase in NO as the CKD stage progressed, data which could implicate the combination of hypoxic vasodilation and the decreased endothelial bioavailability of NO in CKD development. Furthermore, in their study, the authors provided data suggesting a substantial decrease in the SOD enzyme activity in patients with T2DM, which could suggest early injury to glomerular mesangial cells. The activity of the GPx enzyme was significantly increased in the patients with type 2 DM as compared to those without DM. Furthermore, a substantial decrease in the TAC marker was found in the patients with T2DM compared to the patients without DM. A significant increase in the pro-inflammatory cytokines IL-6 and TNF-α was observed in the T2DM group. The authors concluded that in the patients with type 2 DM, oxidative imbalance was characterized by an increase in oxidative markers and a decrease in antioxidant defenses (SOD, TAC, and GPx) [7].

In their review, *Watanabe K*, et al. summarize the recent advances in the mechanisms which contribute to the pathogenesis of DKD, highlighting compelling evidence concerning the molecular mechanisms involved in the condition’s development and progression. The complex pathogenesis of DKD involves various pathways which implicate inflammatory (TNF-alpha, IL-1, IL-6, IL-16, IL-18, MCP-1, and MMP-9), fibrotic (transforming growth factor-beta, fibronectin, collagen-1, and connective tissue growth factor), metabolic (reactive oxygen species, advanced glycation end-products, gut microbiome changes), and hemodynamic factors (angiotensin II, aldosterone, endothelin), respectively. In addition, attention is paid to the recent advances in treatments for DKD, such as angiotensin-converting enzyme inhibitors, angiotensin II receptor blockers, SGL2i, mineralocorticoid receptor antagonists, glucagon-like peptide-1 receptor agonists, and endothelin receptor antagonists [8].

Gut dysbiosis and microbiota-derived metabolites are involved in the early pathophysiological mechanisms of DKD. Microvascular complications that derive from metabolic changes may be related to gut-derived biomarkers. To date, the gut–renal–cerebral axis has been majorly implicated in the occurrence of severe complications in the course of type 2 DM [9,10]. *Balint L*, et al. studied the complex interconnection of gut-derived biomarkers with podocyte injury, proximal tubule dysfunction, and renal and cerebrovascular endothelial dysfunction. The study design consisted of the metabolite profiling of the serum and urine of 90 T2DM patients (subgroups P1—normoalbuminuria, P2—microalbuminuria, P3—macroalbuminuria) and 20 healthy controls (group C), based on ultra-high-performance liquid chromatography coupled with electrospray ionization-quadrupole-time of flight-mass spectrometry analysis (UHPLC-QTOF-ESI+-MS). Through multivariate and univariate analyses of the serum and urine, which included Partial Least Squares Discriminant Analysis (PLSDA), Variable Importance Plots (VIPs), Random Forest scores, One Way ANOVA, and biomarker analysis, there were discovered metabolites belonging to the nitrogen metabolic pathway and retinoic acid signaling pathway which differentiated the P1 group from the P2, P3, and C groups. Tyrosine, phenylalanine, indoxyl sulfate, serotonin sulfate, and all-trans retinoic acid comprised the metabolic fingerprint of the P1 group vs. the P2, P3, and C groups, revealing a particular pattern in early DKD in the T2DM patients. The authors summarize their findings and point to the fact that the metabolomic biomarkers found in their study were derived from phenylalanine, tyrosine, tryptophan, and retinoic acid metabolism and revealed a specific metabolic fingerprint in the normoalbuminuric type 2 DM patients. Furthermore, the dynamics of these gut-derived metabolites may highlight a common pathogenic pathway within the kidney and the brain with regard to podocyte injury, proximal tubule dysfunction, and renal and cerebral vascular structures. The metabolite profiling of the gut–renal–cerebral axis revealed a particular pattern in early DKD in the T2DM patients, even in the normoalbuminuric stage [11].

Dipeptidyl peptidase-4 inhibitors have anti-inflammatory effects that provide endothelial protection. Protein tyrosine phosphatase (PTP) 1B (PTP1B) is one of the most important PTPs, and displays dual regulatory activity that acts upon various signaling pathways, and controls glucose uptake and insulin regulation. Also, PTP1B regulates the pro-inflammatory and anti-inflammatory effects of the Janus kinase-signal transducer and activator of transcription (JAK-STAT) signaling pathways in hematological diseases. In their experimental study conducted on rats, *AL-Qabbaa SM*, et al. proposed the hypothesis that DPP-4 inhibition is associated with the PTP1B/JAK-STAT pathway, enabling the alleviation of DN. The authors studied the therapeutic effects of sitagliptin, a DPP-4 inhibitor, on DKD and its potential underlying mechanisms of action. The levels of inflammatory biomarkers, such as protein tyrosine phosphatase 1 B (PTP1B), phosphorylated Janus kinase 2 (P-JAK2), and phosphorylated signal transducer activator of transcription (P-STAT3), in kidney tissues were assessed and kidney sections were examined histologically. The renoprotective effects of DPP-4 inhibitors found in this study were expressed through the attenuation of inflammation via the modulation of PTP1B and the JAK/STAT signaling pathway, contributing to the mitigation of DN. The results suggest that sitagliptin can be considered a promising drug for DN treatment. In addition, this study provides compelling evidence for the important role of the PTP1B and JAK/STAT signaling pathways in the inflammation-driven development of DKD [12].

In their study, *Pazarín-Villaseñor L*, et al. attempt an approach to assess the oxidative and antioxidant status in peritoneal dialysis (PD) patients with type 2 DM. The authors found an imbalance in the oxidative status in the patients undergoing PD with and without DM through the significant increase in oxidants, such as lipoperoxides and 8-Iso Prostaglandin F2*a* (8-Isoprostanes (8-IP), and a decrease in nitric oxide.

Superoxide Dismutase (SOD), Glutathione Peroxidase (GPx), Catalase, and the total antioxidant capacity (TAC), as mechanisms of antioxidant defense, were also modified. The activity of the antioxidant enzymes SOD and GPx were significantly increased in the patients with and without DM undergoing PD, possibly in an attempt to compensate for the deregulation of oxidants. In this study, a significant increase in the levels of the oxidative DNA damage marker LPO in the PD patients with and without type 2 DM was also found, as well as the possible inactivity of the DNA repair enzyme. The increase in the activity of the antioxidants SOD and GPx was notable, suggesting a compensatory effect to protect the mitochondrial integrity against oxidative stress presented by the patients with and without type 2 DM undergoing PD. The authors conclude that PD provides better antioxidant protection than other types of RRT in CKD and should be considered the first treatment choice in certain categories of patients in order to provide better cardiovascular protection [13].

Podocyte damage and renal inflammation are interconnected in the pathogenesis of DKD. Pyroptosis is a highly inflammatory form of lytic programmed cell death that occurs most frequently in relation to infection with intracellular pathogens and represents a type of antimicrobial defense mechanism. Lysophosphatidic acid (LPA) is a small bioactive phospholipid which comprises a glycerol backbone with a hydroxyl group, a phosphate group, and a fatty acid chain. The inhibition of lysophosphatidic acid (LPA) receptor 1 (LPAR1) suppresses glomerular inflammation and improves DKD. *Kim D*, et al. investigated LPA-induced podocyte damage and its underlying mechanisms in DKD. The authors investigated the effects of AM095, an orally bioavailable LPAR1-specific antagonist, on podocytes from streptozotocin (STZ)-induced diabetic mice. E11 cells were treated with LPA in the presence or absence of AM095, and the expression of NLRP3 inflammasome factors and pyroptosis was measured. AM095 administration inhibited podocyte loss, NLRP3 inflammasome factor expression, and cell death in the STZ-induced diabetic mice. The effects and mechanisms of blocking LPA/LAPR1 signaling in terms of podocyte damage during DKD were also studied. In the E11 cells, LPA increased NLRP3 inflammasome activation and pyroptosis via LPAR1. The molecular mechanism underlying LPA-induced Egr1 expression in the E11 cells shows that Egr1 may participate in LPA-induced NLRP3 inflammasome activation and pyroptosis in DN. Egr1 mediated NLRP3 inflammasome activation and pyroptosis in the LPA-treated E11 cells. LPA decreased H3K27me3 enrichment at the Egr1 promoter in the E11 cells by downregulating EzH2 expression. EzH2 knockdown further increased LPA-induced Egr1 expression. In podocytes from the STZ-induced diabetic mice, AM095 suppressed Egr1 expression increase and EzH2/H3K27me3 expression reduction. The authors underline that the results of the study show that LPA induces the expression of Egr1 through LPAR1, which activates the NLRP3 inflammasome and induces podocyte damage and death in the progression of DN. The conclusions of the study point to the underlying mechanisms of LPA-induced damage in podocytes during DN progression [14].

The articles published in this Special Issue provide meaningful data derived from experimental and clinical studies. Moreover, the comprehensive reviews sum up recent data from the literature with regard to the intertwining molecular mechanisms involved in the pathogenesis of DKD. To date, the research in the field of DKD has emphasized the importance of an early diagnosis of DKD, with a view to an effective and personalized therapeutic approach to DKD in patients with type 2 DM [15].
